# Adipose Tissue-Derived Signatures for Obesity and Type 2 Diabetes: Adipokines, Batokines and MicroRNAs

**DOI:** 10.3390/jcm8060854

**Published:** 2019-06-14

**Authors:** Min-Woo Lee, Mihye Lee, Kyoung-Jin Oh

**Affiliations:** 1 Soonchunhyang Institute of Medi-bio Science, Soonchunhyang University, Cheonan 31151, Korea; mwlee12@sch.ac.kr (M.-W.L.); mihyelee@sch.ac.kr (M.L.); 2 Metabolic Regulation Research Center, Korea Research Institute of Bioscience and Biotechnology (KRIBB), 125 Gwahak-ro, Yuseong-gu, Daejeon 34141, Korea; 3 Department of Functional Genomics, KRIBB School of Bioscience, Korea University of Science and Technology (UST), 217 Gajeong-ro, Yuseong-gu, Daejeon 34141, Korea

**Keywords:** obesity, type 2 diabetes mellitus, adipokines, batokines, exosomal miRNAs, potential therapeutic targets

## Abstract

Obesity is one of the main risk factors for type 2 diabetes mellitus (T2DM). It is closely related to metabolic disturbances in the adipose tissue that primarily functions as a fat reservoir. For this reason, adipose tissue is considered as the primary site for initiation and aggravation of obesity and T2DM. As a key endocrine organ, the adipose tissue communicates with other organs, such as the brain, liver, muscle, and pancreas, for the maintenance of energy homeostasis. Two different types of adipose tissues—the white adipose tissue (WAT) and brown adipose tissue (BAT)—secrete bioactive peptides and proteins, known as “adipokines” and “batokines,” respectively. Some of them have beneficial anti-inflammatory effects, while others have harmful inflammatory effects. Recently, “exosomal microRNAs (miRNAs)” were identified as novel adipokines, as adipose tissue-derived exosomal miRNAs can affect other organs. In the present review, we discuss the role of adipose-derived secretory factors—adipokines, batokines, and exosomal miRNA—in obesity and T2DM. It will provide new insights into the pathophysiological mechanisms involved in disturbances of adipose-derived factors and will support the development of adipose-derived factors as potential therapeutic targets for obesity and T2DM.

## 1. Introduction

Modern sedentary lifestyle and excessive calorie intake have increased the chance of developing metabolic diseases such as obesity and type 2 diabetes mellitus (T2DM) [1,2,3]. Obesity and T2DM are two of the most pressing public health concerns worldwide because of their association with life-threatening diseases, including cardiovascular diseases and cancers [4,5,6,7]. Obesity, especially pathologic expansion of visceral white adipose tissue (vWAT), increases the risk of developing T2DM. Depending on the race, more than 75–90% of patients with T2DM are overweight or obese. The strong association of obesity and T2DM is supported by the term “diabesity” [8,9]. Obesity and the progression from obesity to T2DM can partly be explained by changes in adipose tissue (AT) composition and function.

The AT is an active endocrine organ secreting several hundreds of bioactive molecules, referred to as adipokines [10,11,12]. The adipokines affect adipocyte functions in an autocrine and paracrine manner and enable the AT to extensively communicate with the brain, liver, muscle, pancreas, and other organs in an endocrine manner [13,14,15]. The AT has been classically considered as a fat reservoir for storage of excess calories. However, it has recently been reported that AT also functions as an energy-consuming organ that participates in the regulation of thermogenesis, the process that dissipates energy in the form of heat [16,17]. The AT is broadly classified into the two depots based on their morphology and functions–white adipose tissue (WAT) and brown adipose tissue (BAT) [18,19]. WAT contains cells with a single large lipid droplet, called unilocular cells that are dynamically altered in response to the calorie state. Excess calories are stored as triacylglycerol (TG) in lipid droplets (lipogenesis), and the stored TG is broken down into fatty acids and glycerol (lipolysis) for use by other organs when energy is required.

In contrast, BAT dissipates lipids in the form of “heat” via β-adrenergic stimulations or cold exposure. Adipocytes in BAT appear as multilocular cells with small lipid droplets, and have a large number of mitochondria and upregulated mitochondrial uncoupling protein 1 (UCP1), which is embedded in the inner membrane of the mitochondrion and uncouples oxidative respiration from ATP synthesis. BAT is subdivided into two types–classical BAT and inducible BAT–based on their origin, location, and developmental features. Classical BAT is observed in specific regions of the body such as the interscapular region and kidney and constitutively sustains its thermogenic activity without any external stimuli. The inducible BAT, known as brite (brown in white), beige, or brown-like AT is present within the WAT, and its amount and activity are induced by stimuli such as cold exposure or β-adrenergic agonists [20,21].

**Figure 1:**
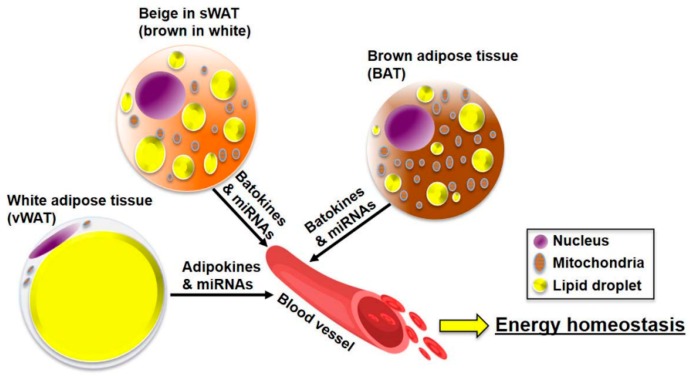
The role of adipose-derived factors (adipokines, batokines, and miRNAs) in the maintenance of energy homeostasis. The adipose tissue (AT) is classified into visceral white adipose tissues (vWAT), subcutaneous white adipose tissues (sWAT), and brown adipose tissue (BAT). The AT secretes adipokines, batokines, and miRNAs into the blood. These adipose-derived factors act like hormones and regulate energy metabolism in tissues, including the brain, liver, AT, muscle, and pancreas.

WAT and BAT secrete bioactive peptides and proteins, referred to as “adipokines” and “brown adipokines or batokines,” respectively [22,23]. It is thought that the WAT-derived adipokines are metabolically different from the BAT-derived batokines, as WAT and BAT differ in morphology and function. Recently, exosomal miRNAs have also been established as factors secreted from ATs [24,25,26]. miRNAs are secreted from AT into the blood and preserved intact inside exosomes. Circulating exosomal miRNAs contain intact genetic information and can reflect the physiological status of ATs. It has been reported that exosomal miRNAs participate in a variety of metabolic processes such as glucose/lipid metabolism, insulin signaling, inflammation, and adipogenesis in various tissues. These adipose-derived signals, including adipokines, batokines, and exosomal miRNA, can be altered by the metabolic status. The altered secretion pattern of adipose-derived factors affects the AT itself as well as other metabolic organs, including the brain, liver, muscles, and pancreas (Figure 1).

This review will classify adipokines, batokines, and microRNA as adipose tissue-derived factors, and introduce them as molecular markers of obesity and T2DM.

## 2. Adipokines—Good or Bad?

WAT is the primary organ that stores excess energy in the form of fats. It has been known as an endocrine organ that secretes bioactive peptides and proteins, referred to as adipokines [16]. Therefore, changes in the concentration of adipokines and inflammatory markers can be indicators of AT dysfunction and pathogenic conditions [27,28,29]. Additionally, it provides critical clues that can explain the pathogenic mechanisms of obesity and T2DM. WAT is divided into two regional and functional depots—vWAT and subcutaneous white adipose tissues (sWAT) [30]. vWAT is related to insulin resistance, inflammation, dyslipidemia, obesity, and T2DM caused by the pathogenic expansion of WAT. Conversely, sWAT is frequently associated with metabolic improvement and insulin sensitivity, as it contains brown-like cells known as beige adipocytes or inducible brown adipocytes that perform mitochondrial and thermogenic functions and burn fats. Besides adipocytes, the macrophages, neutrophils, foam cells, endothelial cells, and fibroblasts also function as secretory cells in the AT [31,32]. Therefore, their composition and distribution in AT are also important for the maintenance of metabolic homeostasis.

**Figure 2:**
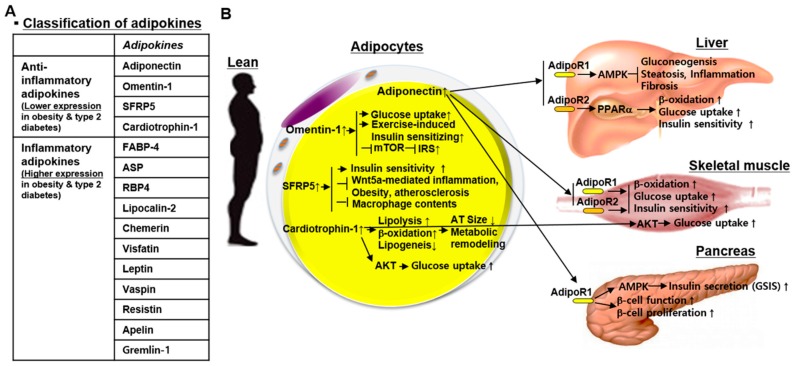
Classification of adipokines and anti-inflammatory adipokines. (**A**) Adipokines are categorized into anti-inflammatory adipokines and inflammatory adipokines, based on their expression in obesity and type 2 diabetes mellitus. (**B**) Anti-inflammatory adipokines, adiponectin, omentin-1, SFRP5, and cardiotrophin-1 improve energy metabolism in the liver, skeletal muscle, and pancreas as well as the adipose tissue itself. Abbreviations: SFRP5, secreted frizzled-related protein 5; FABP-4, fatty acid binding protein 4; ASP, acylation-stimulating protein; RBP4, retinol-binding protein 4; mTOR, mammalian target of rapamycin; IRS, insulin receptor substrate; Wnt5a, wingless-type MMTV integration site family member 5A; AT, adipose tissue; AdipoR, adiponectin receptor; AMPK, AMP-activated protein kinase; PPARα, peroxisome proliferator-activated receptor alpha; GSIS, glucose-stimulated insulin secretion.

**Figure 3:**
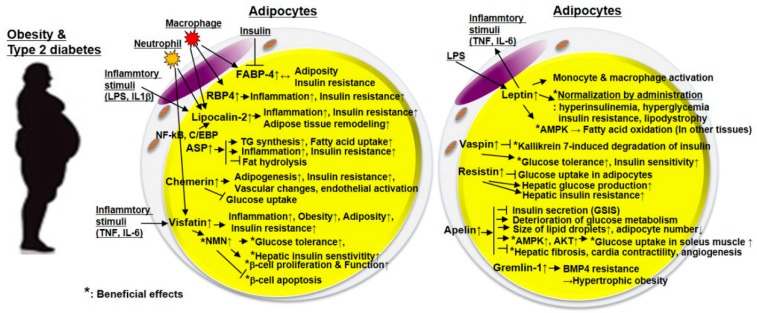
The role of inflammatory adipokines in obesity and type 2 diabetes mellitus (T2DM). There are increased inflammatory adipokines depending on adiposity or obesity. They exacerbate inflammation, insulin resistance, and glucose/insulin metabolism in adipose tissues and other peripheral tissues such as the liver, muscle, pancreas, and blood vessels. In particular, FABP, ASP, RBP4, and lipocalin-2 are correlated with inflammation, obesity, and insulin resistance. Although its levels are increased in obesity and T2DM, vaspin has metabolically beneficial effects as it is thought to compensate for obesity and T2DM. Additionally, leptin, a well-known inflammatory adipokine, exhibits an inflammatory phenotype in adipocytes and inflammatory cells, while administration of leptin improves hyperinsulinemia, hyperglycemia, insulin resistance, glucose/lipid metabolism. Abbreviations: FABP-4, fatty acid binding protein 4; RBP4, retinol-binding protein 4; ASP, acylation-stimulating protein; LPS, lipopolysaccharides; IL, interleukin; NF-κB, nuclear factor kappa light chain enhancer of activated B cells; C/EBP, CCAAT-enhancer-binding protein; TNF, tumor necrosis factor; NMN, nicotinamide mononucleotide; AMPK, AMP-activated protein kinase; GSIS, glucose-stimulated insulin secretion; BMP4, bone morphogenetic protein 4.

In this section, we consider bioactive molecules derived from all secretory cells in the WAT, as adipokines. Adipokines can be broadly classified into anti-inflammatory and inflammatory adipokines, depending on their expression levels under obese conditions—adipokines that are upregulated under obese conditions are categorized as inflammatory adipokines (Figure 2A). An extensive review on a large number of adipokines has been published by Oh et al. [13]. Therefore, in this review, we have introduced new adipokines along with traditional adipokines. We have briefly summarized pathological changes in adipokine expression and discussed the usefulness and clinical significance of adipokines as therapeutic targets for obesity and T2DM (Figure 2 and Figure 3). Adipokines, such as FGF21, that have thermogenic functions and are derived from beige adipocytes in sWAT are categorized into batokines. These adipokines will be described in a different section on batokines.

### 2.1. Anti-Inflammatory Adipokines

#### 2.1.1. Adiponectin

Adiponectin is one of the most abundant adipokines that is highly expressed in WAT and has anti-obesity, anti-atherogenic, and anti-diabetic effects [33,34,35]. Patients with obesity and/or T2DM exhibit significantly reduced circulating adiponectin levels [36,37,38]. It is a 30-kDa protein with 244 amino acids. Extensive post-transcriptional modifications facilitate multimerization and secretion of adiponectin [39,40,41]. After post-transcriptional modifications, adiponectin is secreted into the blood in three different homomeric complexes—trimer (the low-molecular-weight (LMW) form), hexamer (the medium-molecular-weight (MMW) form), and multimer (the high-molecular-weight (HMW) form). Among them, the HMW form contributes to favorable metabolic effects of adiponectin correlated with glucose tolerance, insulin sensitivity, and weight loss [42,43]. In the liver, adiponectin alleviates steatosis, fibrosis, and inflammation [44,45]. In the pancreatic islet β-cells, adiponectin enhances glucose-stimulated insulin secretion (GSIS) via AMPK activation, and increases β-cell function and proliferation [46,47]. Skeletal muscles can also generate and secrete adiponectin [48]. It is thought that adiponectin has insulin-sensitizing and anti-diabetic functions in skeletal muscles. Adiponectin signals via two adiponectin receptors—AdipoR1 and AdipoR2—on the target cells [49,50,51]. AdipoR1 and R2 were first cloned in 2003. AdipoR1 is expressed in several tissues, including skeletal muscles, heart, spleen, kidney, and liver. AdipoR2 is mainly expressed in the liver. AdipoR1 is associated with AMPK activation and suppression of gluconeogenic and lipogenic gene expression. AdipoR2 is related to activated PPARα, increased glucose uptake, enhanced fatty acid oxidation, and improved insulin sensitivity. Both AdipoR1 and R2 are involved in glucose/lipid metabolism and insulin sensitivity. Importantly, circulating adiponectin levels are inversely correlated with obesity, diabetes, and obesity-related diseases [36,37,38]. Therefore, adiponectin is a promising and attractive target for the treatment of obesity and T2DM.

#### 2.1.2. Omentin-1

Omentin-1, also known as intelectin-1, has been recently identified as a novel adipokine consisting of 313 amino acids that is selectively produced by the vWAT [52]. It exerts anti-inflammatory, anti-obesity, anti-diabetic properties, and has beneficial effects on exercise-induced insulin-sensitization [53,54,55,56,57]. The AT consists of two major components – the mature adipocytes and the stromal vascular fraction containing preadipocytes, macrophages, lymphocytes, and endothelial cells [58,59]. Omentin-1 is abundantly expressed in the stromal vascular fraction of the vWAT [60,61]. It is also expressed at extremely low levels in the sWAT and mature adipocytes of vWAT [60]. In human adipocytes, omentin-1 enhanced insulin-stimulated glucose uptake and activated insulin receptor substrate (IRS) by inhibiting the mTOR signaling pathway [60,62]. Omentin-1 levels are significantly decreased in the serum and vWAT of overweight and obese individuals as well as in the serum of T2DM patients [53,54,55,56]. Collectively, omentin-1 as an adipokine could be a new marker for predicting the risk of metabolic diseases such as obesity and T2DM.

#### 2.1.3. Secreted Frizzled-Related Protein 5 (SFRP5)

Secreted frizzled-related protein 5 (SFRP5) has been newly identified as an adipokine that is predominantly expressed in WAT rather than in other ATs. It has beneficial effects on insulin sensitivity and inflammation and is known as a novel effector of adipose tissue-linked chronic inflammation [63]. It was reported as a negative regulator that inhibits Wnt signaling transduction by binding to Wnt protein [64]. Reduced Srfp5 provoked Wnt5a-mediated inflammation, obesity, and atherosclerosis [63,65]. Additionally, depletion of SFRP5 augmented the macrophage population, and the proinflammatory proteins in mouse AT, and exhibited impaired glucose clearance and reduced insulin sensitivity [63]. Serum SFRP5 levels are low in subjects with obesity and T2DM [66,67,68,69]. These demonstrate that decreased expression of SFRP5 is a prognostic marker for the risk of obesity and T2DM.

#### 2.1.4. Cardiotrophin-1

Cardiotrophin-1 (CT-1) was identified as a member of the IL-6 cytokine family due to its structural similarity with other IL-6 family members [70]. It is considered as a key regulator of glucose and lipid metabolism, and as a factor that can improve obesity and insulin resistance [71,72]. CT-1 is expressed at high levels in liver, kidney, skeletal muscle, heart, and lung, and is expressed at low levels in the brain and testis [73]. CT-1 is chiefly produced and released from the AT [74]. CT-1 deficient mice showed obesity, insulin resistance, and high levels of cholesterol in the blood, despite decreased food intake [71]. These findings demonstrate that loss of CT-1 is associated with lower energy expenditure. Conversely, chronic administration of CT-1 reduced the size of adipocytes through decreased expression of lipogenic genes and promoted the expression of genes related to lipolysis and fatty acid oxidation, subsequently leading to metabolic remodeling of WAT in mice [71]. Additionally, CT-1 improved glucose homeostasis and promoted glucose uptake by insulin-stimulated phosphorylation of AKT in myotubes and adipocytes [71]. Further, acute and chronic administration of CT-1 exhibited hypoglycemic and anti-obesity properties through low intestinal sugar uptake [75]. CT-1 exerted lipolytic properties in AT through the activation of cAMP/protein kinase A (PKA) signaling pathway [76]. The effects of CT-1 levels on obesity and metabolic diseases in humans is controversial. However, many studies described that expression levels of CT-1 are decreased in WAT of obese mice, and significantly reduced in overweight and obese subjects [77,78,79,80].

### 2.2. Inflammatory Adipokines

#### 2.2.1. Fatty Acid Binding Protein 4 (FABP-4)

Fatty acid binding protein 4 (FABP4; also known as aP2) is mainly expressed in adipocytes and macrophages [81]. It plays a critical role in insulin resistance, atherosclerosis, and inflammation. FABP4 is released from adipocytes, and its level is relatively higher than other adipokines in the serum [82]. Circulating FABP4 levels are elevated in obesity-related metabolic disorders such as obesity, insulin resistance, diabetes, hypertension, atherosclerosis, and impaired myocardial contractility [83,84,85]. Further, circulating levels of FABP4 is positively correlated with adiposity shown by body mass index (BMI) and body fat percentage, whereas it is negatively correlated with plasma concentration of adiponectin [86]. Obesity increases insulin secretion from pancreatic β-cells. FABP4 promotes insulin secretion in vitro and in vivo, and high circulating levels of FABP4 promotes GSIS in humans [87]. Conversely, insulin suppresses FABP4 secretion from adipocytes in vitro and in mice and humans [87,88]. These data suggest that FABP4 increases insulin secretion from pancreatic β-cells during obesity, as an adaptive β-cell response with adiposity, suggesting that FABP4 is a detectable proinflammatory biomarker that can mirror adiposity and obesity.

#### 2.2.2. Acylation-Stimulating Protein (ASP)

ASP is an adipocyte autocrine factor that increases triglyceride synthesis from glucose and free fatty acids (FFA) in adipocytes [89]. Several studies described that plasma ASP levels are increased in obese and hyperlipidemic individuals, and that increased ASP levels are positively correlated with increased TG levels [90,91,92,93]. Conversely, weight loss and fasting, conditions that lower TG, reduced ASP levels [91,93]. Further, administration of ASP enhanced high-fat diet (HFD)-induced insulin resistance by increased adipocyte inflammatory response [94]. Insulin increases fatty acid uptake by adipocytes and inhibits fat hydrolysis from adipocytes. ASP plays its role in an additive and independent manner to the action of insulin [95]. Additionally, ASP is a cleavage product of complement 3 (C3), a critical component of the innate immune system [94]. Therefore, the lack of C3 results in ASP deficiency and consequently leads to decreased leptin levels and reduced body fat [94]. Furthermore, loss of ASP contributed to delayed clearance of postprandial triglycerides and fatty acids [96]. These data demonstrate that ASP has powerful anabolic effects on AT.

#### 2.2.3. Retinol-Binding Protein 4 (RBP4)

Retinol-Binding Protein 4 (RBP4) is a 21 kDa hepatocyte-secreted factor and a retinol transporter [97]. Recently, it was reported that RBP4 is also secreted by adipocytes and macrophages as well as the liver [98,99]. RBP4 concentrations are associated with high levels of blood pressure, cholesterol, triglycerides, and BMI, and are related to low levels of high-density lipoprotein (HDL) [100]. Serum RBP4 levels are highly expressed under insulin resistance conditions related to obesity and T2DM [98,100]. RBP4 is more markedly expressed in visceral fat rather than subcutaneous fat, and RBP4 expression in visceral fat is significantly increased in individuals with obesity and T2DM [101]. Serum RBP4 levels are positively linked with adipose RBP4 mRNA levels and intra-abdominal fat mass. Adipose RBP4 mRNA levels, especially in the visceral fat, are inversely correlated with GLUT4 mRNA and are positively linked with adiposity in insulin resistance [101]. Treatment of RBP4 suppressed insulin-stimulated phosphorylation of insulin receptor substrate 1 (IRS1) and ERK1/2 in human adipocytes [102]. Furthermore, RBP4 led to insulin resistance by induction of CD4+ T-helper cell polarization and AT inflammation [103]. The lowering of inflammatory RBP4 levels might be a promising target for the treatment of insulin resistance and obesity-related diseases [104].

#### 2.2.4. Lipocalin-2 (LCN2)

Lipocalin-2 (LCN2) is abundantly expressed in the ATs. LCN2, also known as neutrophil gelatinase-associated lipocalin (NGAL), is also expressed in the liver, kidney, lung, and the macrophages and neutrophils [105,106,107,108]. It promotes inflammation, obesity, and insulin resistance, and plays a role in AT remodeling during HFD-induced obesity [109,110,111,112,113]. Expression and secretion of LCN2 are induced by several inflammatory stimuli such as lipopolysaccharides (LPS) and IL1β [106,114]. In particular, the proinflammatory transcription factor NF-κB and CCAAT/enhancer-binding protein (C/EBP) binds to its consensus motif on LCN2 promoter and regulates LCN2 expression [110,115]. LCN2 expression was increased in AT and serum of obese individuals as well as genetic and diet-induced obese mice [110,112,116]. Serum levels of LCN2 correlate with obesity and BMI, especially in severely obese women [117]. Furthermore, LCN2-deficiency attenuated aging- and obesity-induced insulin resistance by inhibiting 12-lipoxygenase, an enzyme that metabolizes arachidonic acids, and tumor necrosis factor-α (TNF-α), a critical insulin resistance inducer [118]. Conversely, some studies showed that depletion of LCN2 in mice increased diet-induced obesity, insulin resistance, and increased expression of proinflammatory mediators, because of impaired thermogenesis [119]. It was further corroborated by another study that showed that LNC2 regulated oxidative metabolism and BAT activation in an adrenergic-independent manner [120]. The reasons for these two conflicting opinions in LNC2-deficient mice is currently unknown. However, it is thought that the beneficial effects of LCN2 might be limited to thermogenesis or BAT metabolism in mice.

#### 2.2.5. Chemerin

Chemerin has been identified as a novel adipokine that regulates adipogenesis and adipocyte metabolism and promotes insulin resistance [121,122,123]. It is abundantly expressed in ATs, liver, and innate immune cells [121,122,123]. Chemerin levels are elevated in serum and ATs of patients with obesity and T2DM and obese and T2DM mouse models such as *ob*/*ob* and *db*/*db* mice [124,125,126,127,128]. Chemerin plays a role in the activation of endothelial molecules (ICAM-1 and E-selectin), atherosclerotic vascular changes, and the pathogenesis of cardiovascular disease in T2DM [127,129]. Further, serum chemerin levels are correlated with abdominal visceral fat accumulation, BMI, blood pressure, insulin resistance, and cholesterol levels [130]. Elevated circulating chemerin impaired glucose tolerance. Consistently, injection of recombinant chemerin aggravated glucose intolerance, reduced serum insulin levels, and impeded tissue glucose uptake in mouse models of obesity and diabetes [126,128]. These observations suggest that chemerin is metabolically harmful and promotes insulin resistance. In contrast, chemerin binds to orphan G protein-coupled receptor CMKLR1 (ChemR23 or DEZ) and regulates intracellular signaling. Chemerin and its receptor ChemR23 promoted insulin-dependent glucose uptake in ATs [131]. Loss of ChemR23 increased LPS-induced inflammatory neutrophil infiltration, because of the reduction in chemerin response [132]. In pancreatic β-cells, chemerin, and its receptor ChemR23 enhanced β-cell function and GSIS, and improved glucose tolerance [133]. These anti-inflammatory effects of chemerin appear to be in a ChemR23-dependent manner.

#### 2.2.6. Visfatin/PBEF/Nampt

Visfatin was identified as a pre-B-cell colony-enhancing factor 1 (PBEF) and a growth factor for B cell precursors in the liver, skeletal muscle, and bone marrow [134]. Circulating visfatin levels reflect the WAT mass and adiposity, and are dependent on insulin resistance [135,136,137]. Its concentration is markedly increased in T2DM patients concomitant with obesity. Further, visfatin is not only produced by ATs, but also by the neutrophils [138]. Individuals with inflammatory diseases have elevated levels of circulating visfatin [139]. It has been shown that visfatin and inflammatory factors influence each other. Visfatin transcription was influenced by TNF, IL-6, and glucocorticoids [139]. Visfatin also stimulated secretion of TNF, IL-6, and IL-1β, and triggered macrophage differentiation, and monocyte-induced alloresponses in lymphocytes [140,141]. These suggest that visfatin mediates proinflammatory signaling. Conversely, visfatin plays a critical role in the synthesis of nicotinamide mononucleotide (NMN) as a phosphoribosyl transferase enzyme ((nicotinamide phosphoribosyltransferase (NAMPT)). Administration of NMN, a product of the NAMPT reaction and a key NAD+ intermediate, improved glucose intolerance and hepatic insulin sensitivity in HFD-induced T2DM. Further, NAMPT-mediated NAD+ biosynthesis ameliorated aging-induced T2DM. These data suggest that NAMPT-mediated NAD+ would contribute to the prevention of obesity and T2DM by high-calorie intake and aging [142]. Additionally, visfatin inhibited β-cell apoptosis, and played a beneficial role in β-cell proliferation and function [143,144]. Collectively, it is thought that visfatin is a proinflammatory mediator, and can exert beneficial effects depending on the physiological and nutritional conditions such as NAMPT-mediated NAD+ biosynthesis by NMN production.

#### 2.2.7. Leptin

Leptin is a classical proinflammatory adipokine that has been identified in *ob*/*ob* mice (*ob* is also known as obese gene or Lep) [145]. Leptin is also known as the satiety hormone that regulates body weight by suppressing hunger. It is a 16-kDa nonglycosylated peptide hormone that is synthesized mainly in adipose cells to regulate weight control via its cognate receptor in the hypothalamus [146]. It has an important role in various cellular process, including reproductive function, fertility, puberty, activity, energy expenditure, atherogenesis, fetal growth, appetite, and food intake [147,148,149]. Leptin, as a well-known obesity marker, enhances the production of TNF and IL-6 in monocytes [150]. It also enhances cell proliferation, the generation of reactive oxygen species (ROS), and migratory responses in monocytes. In macrophages, leptin promotes the production of CC-chemokine ligands by activating the JAK2/STAT3 signaling pathway [151]. Conversely, TNFα mediated lipopolysaccharide (LPS) induced leptin levels, and stimulated leptin secretion from adipocytes [152]. There are two circulating forms of leptin—a biologically active free form and an inactive form that is bound to plasma proteins [153]. The levels of circulating leptin are proportional to the body fat mass both in mice and in humans—obese individuals typically produce higher levels of leptin than lean individuals [154,155]. Leptin levels are correlated with obesity-related diseases such as myocardial infarction and cerebral stroke [156,157]. Leptin stimulated platelet-dependent thrombosis and upregulated vascular adhesion molecules and the prothrombotic tissue factors [158,159]. Further, leptin can be a marker for the levels of energy-dense triacylglycerols in AT. In contrast, leptin deficiency and leptin resistance induce severe insulin resistance [160]. Leptin administration normalized hyperinsulinemia and hyperglycemia and improved insulin resistance and lipodystrophy [160,161,162,163]. Additionally, leptin stimulates fatty acid oxidation and reduces body fat accumulation by activating AMP-activated protein kinase (AMPK) in non-ATs, resulting in improved insulin sensitivity [164,165,166]. These findings demonstrate the ability of leptin to regulate glucose and lipid metabolism and its therapeutic potential in obesity and T2DM.

#### 2.2.8. Vaspin

Visceral adipose tissue-derived serpin (vaspin) is a member of the serine protease inhibitor family [167]. Recently, it was proposed as a useful marker associated with obesity, insulin resistance, and T2DM [168,169,170,171,172]. Human vaspin has been identified as an adipokine of 414 amino acids [167]. It is produced by several tissues, including the AT, liver, skeletal muscle, pancreas, stomach, and skin [173,174,175]. Unlike omentin-1, its expression is not restricted to the vWATs. It is found in 23% of visceral and in 15% of sWATs [172]. Additionally, vaspin is highly expressed in mature adipocytes rather than in the stromal vascular fraction of vWAT [176]. Serum vaspin concentration is high in subjects with obesity and/or T2DM, and is lower in subjects who have a normal body weight than those who are overweight [168,169,170]. The mRNA level of vaspin is enhanced by increased fat mass, impaired glucose tolerance, and decreased insulin sensitivity observed in individuals with obesity and T2DM [172,177]. Many studies support serum vaspin as a potential marker predicting obesity and T2DM. However, these increased levels of vaspin are thought to be compensating for obesity and T2DM [178,179,180,181]. Recombinant vaspin administration improved glucose tolerance and insulin sensitivity [168,182]. Further, it markedly reduced food intake by decreasing the expression of NPY and increasing the expression of POMC in the hypothalamus [183]. The beneficial effects of vaspin in glucose metabolism are closely related to its ability to inhibit protease kallikrein 7, which can cleave and degrade insulin A and B chains [184]. Vaspin inhibits kallikrein 7-induced degradation of insulin, and can consequently stabilize insulin in circulation [184]. Therefore, improved glucose metabolism with vaspin treatment might be associated with the increased circulating insulin concentration. These findings suggest that vaspin is a promising target for the treatment of obesity and T2DM.

#### 2.2.9. Resistin/ADSF

Resistin is a 12.5 kDa cysteine-rich adipose tissue-specific secretory factor (ADSF), which is associated with resistance to insulin [185,186]. It is known as a proinflammatory adipokine. Circulating levels of resistin were increased in genetic and diet-induced obesity and were decreased by treatment with anti-diabetic drug rosiglitazone [185]. Recombinant resistin triggered systemic insulin resistance in mice and decreased insulin-mediated glucose uptake in adipocytes [185]. Contrarily, mRNA levels of resistin are decreased in WAT of obese mice, and administration of anti-resistin antibody improved insulin resistance and blood glucose levels [185,187,188]. It has been reported that infusion of resistin in rodents aggravated hepatic glucose production, and triggered severe hepatic insulin resistance [189]. Under fasting conditions, decreased resistin partly reduced hepatic glucose production by activating AMPK and inhibiting hepatic gluconeogenic enzymes, and regulating hyperglycemia associated with obesity [190]. Further, depletion of resistin in leptin-deficient (*ob*/*ob*) and diet-induced obesity mice improved hepatic glucose production and increased peripheral glucose uptake [191]. Resistin induced suppressor of cytokine signaling 3 (SOCS-3), a known inhibitor of insulin signaling, to regulate glucose metabolism [192]. Although there are exceptions, resistin is thought to induce insulin resistance in mice. Resistin synthesis in mice is limited to adipocytes, whereas in humans, it is expressed at a low level and is produced by macrophages and monocytes, but not adipocytes [193]. The protein sequence and expression pattern of resistin in humans is different from those in rodents. It is still unclear whether resistin promotes insulin resistance in humans [185,194,195,196].

#### 2.2.10. Apelin

Apelin has been identified as an adipokine that contributes to the regulation of glucose metabolism in an endocrine fashion [197,198]. It is an endogenous ligand of the G-protein-coupled receptor and is expressed in various tissues, such as the skeletal muscles, heart, stomach, and central nervous system (especially in the hypothalamus) as well as in the ATs [199,200]. Higher circulating apelin is observed in patients with obesity and T2DM and is associated with insulin resistance and hyperinsulinemia in vivo and in vitro [197,201,202,203]. Apelin-induced deterioration of glucose metabolism mediated by impeding GSIS [202]. Increased circulating apelin inhibited insulin release and impeded glucose metabolism in mice. Further, apelin decreased adipocyte number and increased the size of lipid droplets by controlling activation of G(q), G(i), and AMPK, suggesting that apelin would negatively regulate lipolysis in ATs [204,205]. Conversely, apelin promoted glucose uptake by the activation of the AMPK and AKT signaling pathway in soleus muscle [206]. In obese and insulin-resistant models, administration of apelin improved glucose tolerance and glucose utilization, indicating that elevated circulating apelin might be a phenotype of apelin resistance or an adaptation of the body to raised apelin levels [206]. Moreover, many studies reported the beneficial effects of apelin on hepatic fibrosis, cardiac contractility, blood pressure, cardiovascular and fluid homeostasis, food intake, cell proliferation, and angiogenesis [203,207,208]. Further studies are required to understand the effects of apelin under physiological and pathological conditions.

#### 2.2.11. Gremlin-1

Gremlin is a glycoprotein that belongs to the Dan (neuroblastoma) family [209]. It exerts an inhibitory effect by forming heterodimers with bone morphogenetic protein (BMP) that belong to the transforming growth factor-beta (TGF-β) family [210,211]. Three alternative splicing forms of gremlin—gremlin1, gremlin2, and gremlin3—have been identified, and gremlin1 is the most common isoform [209]. Gremlin1 is a 184 amino acid (25 kDa) cysteine knot superfamily protein with an eight-membered ring [212]. Gremlin1 secreted from (pre)adipocytes directly binds to BMP4 and prevents its interaction with BMP receptors [212]. BMP4 is involved in the process that determines the fate of adipose precursor cells toward the white adipose lineage and is induced during preadipocyte differentiation in humans [213,214,215,216]. Further, BMP4 expression in WAT of mice led to brown-like changes in sWAT and stimulated white-to-brown transition [215,217]. Hypertrophic obesity is associated with AT dysfunction and insulin resistance and is partly caused by impaired subcutaneous adipogenesis [216]. Gremlin1 is increased in hypertrophic obesity in humans, a state of BMP4 resistance. It is thought that increased Gremlin1 partly contributes to BMP4 resistance in hypertrophic obesity [218,219]. Further, the effect prominently appeared in beige/brown adipogenesis rather than white adipogenesis.

## 3. Batokines

**Figure 4:**
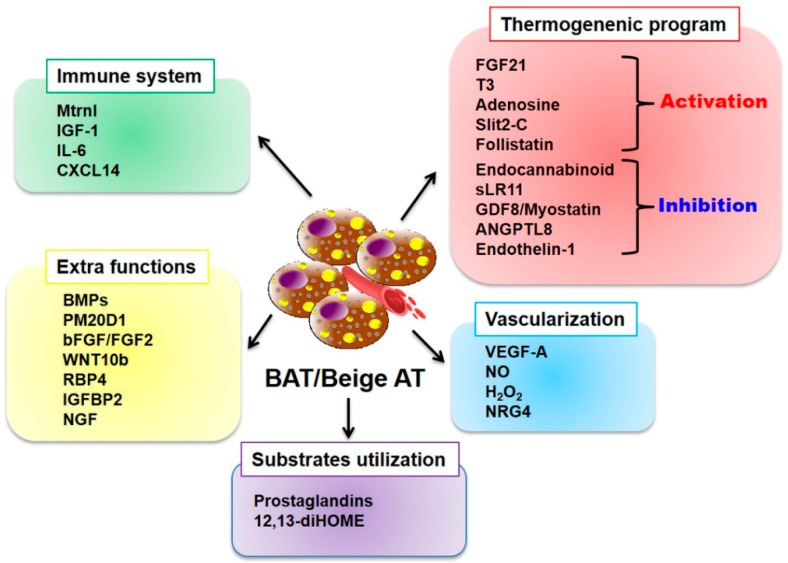
Batokines secreted from BAT and beige AT. Batokines secreted from BAT and beige AT contribute to the regulation of various functions such as thermogenic activity, immune activity, vascularization, substrate utilization, and other functions. Abbreviations: Mtrnl, meteorin-Like; IGF-1, insulin growth factor-1; IL-6, interleukin-6; CXCL14, chemokine (C-X-C motif) ligand 14; BMPs, bone morphogenetic proteins; PM20D1, peptidase M20 domain containing 1; bFGF, basic fibroblast growth factor; WNT10b, wingless-Related MMTV Integration Site 10b; RBP4, retinol-binding protein-4; IGFBP2, insulin-like growth factor-binding protein-2; NGF, nerve growth factor; 12,13-diHOME, 12,13-dihydroxy-9Z-octadecenoic acid; FGF21, fibroblast browth factor 21; T3, triiodothyronine; sLR11, soluble form of the low-density lipoprotein receptor relative LR11; GDF8, growth differentiation factor-8; ANGPTL8, angiopoietin-like8; VEGF-A, vascular endothelial growth factor A; NO, nitric oxide; H_2_O_2_, hydrogen peroxide; NRG4, neuregulin-4.

Beyond the well-known classical function of storing excess energy in the form of triacylglycerol, adipocytes are also involved in dissipating energy in the form of non-physical matter, heat. Since the discovery of the thermogenic function of adipocytes, it has been suggested that these heat-producing adipocytes are broken down into two groups—classical brown adipocytes and brown-like (also known as beige or brite) adipocytes based on their origin, location, and developmental features. In addition, even though they belong to the AT and have a profile that is different from the WAT, BAT and beige WAT can secrete several peptides and non-peptides. These secretory factors enhance the thermogenic activity by regulating hypertrophy and hyperplasia in adipocytes and also help to attain maximum activity by regulating vascularization, innervation, and substrate utilization in ATs, processes that are all integrally required when exposed to a prolonged cold environment. In this section, we have included factors that are specifically secreted from BAT or beige WAT—the so-called batokines—based on their functional effects on targets (Figure 4).

### 3.1. Regulation of Thermogenic Programs

#### 3.1.1. Fibroblast Growth Factor 21 (FGF21)

Several batokines have been considered to positively or negatively contribute to heat production by regulating thermogenic signaling and gene regulation. FGF21 is one of the well-elucidated batokines, and since its discovery, its beneficial effects have been extensively studied in both rodents and humans. FGF21 belongs to the FGF family and is considered to regulate glucose and lipid metabolism and energy homeostasis. It is mainly expressed in the liver and WAT [220,221]. It was recently reported that besides the liver and WAT, BAT and beige WAT also contribute to systemic FGF21 levels [222,223,224]. When exposed to cold or its relevant stimuli such as norepinephrine or CL-316243, FGF21 expression and secretion by BAT or beige WAT is upregulated through the β3-adrenergic signaling cascade [222,223,224]. Increased FGF21 augments energy catabolism by promoting glucose uptake and oxidation by Glut1 expression and activation of lipolysis and reduction of fatty acid synthesis [225,226]. In contrast, it was demonstrated that FGF21 also directly promotes UCP1-dependent thermogenesis in BAT and beige WAT [224]. Because of its promising benefits against metabolic disorders, the effects of FGF21 have been re-assessed in humans. It has been shown that FGF21 is preferentially expressed in human beige adipocytes [227]. Further, mild cold exposure increases plasma FGF21 levels with BAT activation in the neck and shoulders [226]. Moreover, neonates have a large amount of BAT content and maintain high levels of FGF21 [228]. These strongly suggest that FGF21 expression in humans is closely associated with the thermogenic activity of ATs. However, despite many studies, it remains to be investigated whether thermogenically active adipocytes are key providers of circulating FGF21 and whether FGF21 produced and secreted during thermogenic activation has similar effects in humans and rodents.

#### 3.1.2. Triiodothyronine (T3)

T3 is a circulating thyroid hormone, which contributes to many physiological processes, including metabolism, thermogenesis, development, and heart rate. Most T3 in circulation is derived from direct release from the thyroid gland and T4 conversion in the liver, hypothalamus and anterior pituitary, each accounting for approximately 20% and 80%, respectively [229]. In the context of cold exposure, T3 is required for the complete activation of thermogenesis. Circulating T3 has a limited function in thermogenesis; in contrast, T3 locally produced from T4 by D2 deiodinase (DIO2), induced by beta-adrenergic signaling in brown or white adipocytes is critical for the induction of thermogenesis by acting through the T3 receptor beta (TRβ1) isoform [230,231,232,233]. However, it remains unknown whether T3 produced in brown or beige adipocytes can act in an endocrine manner.

#### 3.1.3. Adenosine

Recently, it was suggested that purinergic signaling activated by extracellular nucleotides or their derivatives is involved in many physiological and pathological processes of ATs [234]. In particular, adenosine release can be enhanced by sympathetically activated brown adipocytes, and this released adenosine positively contributes to the activation of BAT and browning of WAT in humans and rodents, suggesting that it is a potential therapeutic target against diet-induced obesity [235].

#### 3.1.4. Slit2-C

Slit is a secreted extracellular matrix protein, which was first identified in the development of the central nervous system in Drosophila. Slit consists of three homologs—Slit1, Slit2, and Slit3. Under thermogenic stimulation, in mammals, it has been unraveled that Slit2 can be secreted in a cleaved form, called Slit2-C, from beige adipocytes. The secreted Slit2-C can stimulate adipose thermogenesis by activating the PKA signaling cascade, resulting in augmented energy expenditure and improved glucose homeostasis in mice [236]. Furthermore, Kang et al. demonstrated Slit2 presence in human circulation, and its level revealed a negative correlation with diabetic patients [237].

#### 3.1.5. Follistatin

Follistatin (Fst) is a soluble glycoprotein highly expressed in BAT. Exposure to cold induces Fst secretion into the circulation. Fst confers positive effects on brown and beige adipose thermogenesis by blocking the inhibitory cue from TGF-β/Smad3/myostatin signaling [238,239].

#### 3.1.6. Endocannabinoids

When BAT activation or WAT browning appears, synthesis and release of endocannabinoids are induced in BAT and WAT, which then exert inhibitory functions in an autocrine manner [240]. Mechanistically, endocannabinoids bind to the cannabinoid 1 receptor (CB1R) and interrupt the activated β3-adrenoreceptor signaling cascade by suppressing the levels of intracellular cyclic cAMP, thus turning off the BAT activation and WAT browning [240,241].

#### 3.1.7. Soluble form of the Low-Density Lipoprotein Receptor Relative LR11 (sLR11)

sLR11 was identified as one of the negative regulators of thermogenesis, and its expression and serum level increase when thermogenesis is activated. Whittle et al. demonstrated that sLR11 regulates the balance between lipid storage and oxidation in response to changing environmental temperature and diet. Mechanistically, sLR11 binds directly to the BMP receptor, and thus interrupts its downstream signaling, resulting in decreased thermogenesis [242]. It has also been revealed that the sLR11 level positively correlates with the fat mass in humans.

#### 3.1.8. Growth Differentiation Factor-8 (GDF8/Myostatin)

Activation of Agouti-related peptide (AgRP) neurons by an energy deficit promotes the expression of GFP8 in BAT. In turn, in an autocrine manner, GDF8 serves as a negative regulator of brown adipogenesis and thermogenesis through the activation of myostatin/activin receptorIIB (ActRIIB)/Smad3 signaling [243,244].

#### 3.1.9. Angiopoietin-Like8 (ANGPTL8)

ANGPTL8, also called lipasin, RIFL (refeeding induced fat and liver) or betatrophin, is expressed in the liver, WAT, and BAT. A study suggested that ANGPTL8 represses the activity of lipoprotein lipase, a protein that stimulates lipolysis [245]. With regard to thermogenesis, although it has not yet been demonstrated, it is assumed that ANGPTL8 negatively regulates the thermogenic process. In line with this, Fu et al. showed that cold exposure dramatically increased ANGPTL8 expression in BAT of mice [246]. In humans, as ANGPTL8 expression is only detectable in the WAT [247], the question of whether ANGPTL8 expression in WAT is altered by cold-stimulation remains open.

#### 3.1.10. Endothelin-1

Endothelin-1 is initially expressed in a precursor form, referred to as preproendothelin-1 (PPET1), in the vascular endothelial cells, and when proteolytically cleaved, it likely contributes to blood vessel constriction. Endothelin-1 (ET-1) is expressed and secreted by brown and beige adipocytes. Released ET-1 inhibits brown and beige adipogenesis via G(q) signaling. Moreover, ET-1 secretion by both cells can be blocked by the activation of β3-adrenergic signaling, likely acting as a negative regulator for thermogenesis [248].

### 3.2. Regulation of Vascularization in Adipose Tissues

#### 3.2.1. Vascular Endothelial Growth Factor A (VEGF-A)

Appropriate regulation of vasculature is an essential process in the maintenance of BAT and WAT functions under an altered environment, temperature, or diet [249]. In particular, during cold acclimation, vascularization in both BAT and WAT is markedly enhanced in order to increase the supply of nutrients and oxygen and to export the generated heat to the periphery. It is known that vascularization in AT is in part, dependent on VEGF-A signaling [250,251]. As direct evidence for the significance of VEGF-A-vascularization axis in thermogenesis, specific overexpression of VEGF-A in adipocytes promotes vascularization and UCP1 expression both in WAT and BAT, enhancing thermogenesis. Conversely, depletion of VEGF-A in the adipocytes of normal mouse inhibited thermogenesis, suggesting that VEGF-A-induced vascularization is a key step in the activation of thermogenesis [252,253]. Importantly, Shimizu et al. proposed VEGF-A as a potential therapeutic intervention for obesity-associated insulin resistance. Introduction of VEGF-A into AT of the obese mouse could recover vascularity and impaired insulin sensitivity and systemic glucose metabolism [253].

#### 3.2.2. Nitric Oxide

Under cold environments, nitric oxide (NO) makes two important contributions to thermogenic adaptation [254]. NO not only enhances the vasodilation in BAT, but also increases the expression of key thermogenic components such as PGC1-a, cell death-inducing DFFA-like effector A (CIDEA) and UCP1 in white adipocytes [255,256]. When NO is produced by inducible nitric oxide synthase (iNOS) in brown adipocytes, it can be released, and in turn, enhances the transfer of substrates and heat by dilating blood vessels [255]. In contrast, NO can activate the thermogenic process in WAT independently of the iNOS pathway. NO is synthesized by serial reduction from inorganic nitrate and nitrite and is known to promote browning of WAT through the activation of the PKG-PGC1-a signaling pathway [256].

#### 3.2.3. Hydrogen Peroxide (H_2_O_2_)

Abnormal vascular remodeling is intimately associated with the development of hypertension in patients with obesity and diabetes. Perivascular ATs (PVATs) of different adipose depots exhibit discrete adipose features—white phenotype at resistance vessels vs. brown phenotype at the aorta [257]. Given these different functional features, PVATs of resistance vessels rather than those of the aorta is prone to be anti-inflammatory and anti-contractile. Recently, Friederich-Persson et al. identified an intriguing mechanism by which H_2_O_2_ determines these two features. Particularly, in BAT or beige AT, H_2_O_2_ produced by NOX4 reduces vascular contractility by activating cyclic GMP-dependent protein kinase G type-1a, raising a possibility that increasing BAT content could recover vascular complications [258].

#### 3.2.4. Neuregulin-4 (NRG4)

Neuregulin-4 (NRG4) is a secretory protein associated with epidermal growth factor (EGF). Although NRG4 is expressed and secreted by all ATs, it is highly enriched in BAT or WAT when exposed to cold [19]. Increased NRG4 has many beneficial effects on metabolic homeostasis mediated in a paracrine or endocrine fashion; these include hepatic lipogenesis, fuel oxidation, nerve innervation, and angiogenesis. NRG4 levels are inversely associated with nonalcoholic fatty liver disease (NAFLD) and type 2 diabetes in rodents [259]. Recently, a similar trend in NRG4 levels was also observed in humans, demonstrating its significance in human diseases. Similar to that in rodents, lower NRG4 levels in circulation are observed in patients with gestational diabetes mellitus, T2DM, metabolic syndrome, and coronary artery diseases [260,261,262].

### 3.3. Regulation of the Immune System in Adipose Tissues

#### 3.3.1. Meteorin-Like (Mtrnl)

Meteorin-like (Mtrnl) was identified as a circulating hormone. Its expression and secretion are induced by exercise and cold exposure in skeletal muscle and AT, respectively. Increased circulating Mtrnl induces a negative energy balance by increasing whole-body energy expenditure and WAT thermogenesis. Mtrnl utilizes an unconventional pathway, regardless of sympathetic nervous system signaling, to activate thermogenesis in beige WAT. Mtrnl stimulates the recruitment of eosinophils and alternatively activated macrophages in WAT, and thus increases the level of catecholamines, the key ligand of β3-adrenergic signaling activation, by promoting secretion from alternatively activated macrophages [263].

#### 3.3.2. Insulin Growth Factor-1 (IGF-1)

IGF-1 is a mitogenic peptide, which is involved in growth, development, and differentiation in various tissues by acting in an endocrine, paracrine, and autocrine manner. Its importance as a batokine was demonstrated by a BAT transplantation trial. When BAT was placed in mice with type 1 diabetes, a significant and sharp increase in the expression of IGF-1 in BAT and circulating IGF-1 levels together with mitigated pro-inflammation and ameliorated glucose homeostasis was observed [264]. In line with this, rats exposed to cold show increased expression of IGF-1, regulating hyperplasia of BAT cells [265].

#### 3.3.3. Interleukin-6 (IL-6)

IL-6 is an interleukin that is produced in response to tissue injury and inflammation and acts as a key mediator of fever and the acute phase response. IL-6 also closely correlates with the development of complications through proinflammatory and autoimmune processes in metabolic diseases such as diabetes and atherosclerosis. In addition, β3-adrenergic stimulation in vitro increases IL-6 expression and secretion by mouse brown adipocytes [266]. Although the detailed mechanism is still unknown, Stanford et al. demonstrated the functional significance of IL-6 present in BAT in vivo. BAT from IL-6 knockout mice transplanted into the visceral cavity of normal mice lead to hepatic inflammation and systemic insulin resistance [267].

#### 3.3.4. Chemokine (C-X-C motif) Ligand 14 (CXCL14)

CXCL14 is a member of the CXC cytokine family. It is known as a potent chemoattractant, enabling immune cells such as monocytes, dendritic cells, and NK cells to localize at inflammatory sites in response to inflammation. In the context of thermogenic activation, BAT secrets CXCL14, which enhances the activation of BAT and induces the browning of WAT by recruiting M2 macrophages [268].

### 3.4. Regulation of Substrate Utilization

#### 3.4.1. Prostaglandins

Several lines of evidence have suggested that prostaglandins and their related catalyzing enzymes are closely associated with BAT activity and browning of WAT. Exposure to cold environment promotes the synthesis and release of prostaglandins, especially PGI_2_ (prostaglandin I_2_), PGE_2_ (prostaglandin E_2_), and a lipocalin prostaglandin D synthase (L-PGDS) in the brown and beige adipocytes [269,270,271,272]. The functional importance of prostaglandins in thermogenic homeostasis was clarified by manipulating the expression of cyclooxygenase 2 (COX2), a rate-limiting enzyme in prostaglandin synthesis, in rodent models. Overexpression of COX2 in white adipocytes positively stimulates the recruitment of beige adipocytes in WAT, while downregulation of prostaglandins by genetic deletion or specific inhibition of COX2 impairs the formation of browning in WAT, which is mediated by shifting adipocyte differentiation from mesenchymal stem cells [273]. Contrarily, Virtue et al. suggested a new concept that prostaglandin-catalyzing enzymes could contribute to BAT activity independent of prostaglandin production. Mice lacking lipocalin prostaglandin D synthase (L-PGDS) showed impaired thermogenic activity in BAT due to a defect in switching of substrate utilization from glucose to lipids [269].

#### 3.4.2. 12,13-Dihydroxy-9Z-Octadecenoic Acid (12,13-diHOME)

Increased lipid production in the body by enhanced lipolysis during cold exposure is an essential process for thermogenesis, as lipids are key fuels utilized to produce heat by oxidation in mitochondria of BAT and beige AT. In addition to this role, Lynes et al. recently proposed that lipid 12,13-dihydroxy-9Z-octadecenoic acid (12,13-diHOME) concentration is increased in the circulation in humans and mice when exposed to cold, which results in the stimulation of fatty acid uptake by promoting the translocation of CD36 and FATP1 to the cell membrane of BAT and beige WAT [274]. In line with this function in ATs, lipid 12,13-diHOME levels are also increased in the circulation after exercise in humans and mice, contributing to the uptake of circulating fatty acids in skeletal muscles [275].

### 3.5. Regulation of Additional Functions in Adipose Tissues

#### 3.5.1. Bone Morphogenetic Proteins (BMPs)

BMPs, a group of TGF-β superfamily members, are important in the development of bone, cartilage, the nervous system, and heart. Many lines of evidence have demonstrated that BMPs are essential morphogens in embryogenesis and development [276]. Among them, BMP2, BMP4, BMP7, and BMP8B have been especially acknowledged to contribute to the regulation of AT development in different locations at different times during development [277]. BMP2 stimulates the commitment into white adipocyte differentiation, and its expression in AT positively correlates with obesity and diabetes in patients. BMP4 shows several essential roles in adipocyte development. First, BMP4 induces the commitment of multipotent mesenchymal stem cells to differentiate to the adipocyte lineage; second, BMP4 drives the committed stem cells into white adipocytes; and lastly, BMP4 contributes to the conversion of brown adipocytes to white adipocytes in BAT [215,217,278,279]. In 2008, Tseng et al. identified a new role of BMP7 in adipogenesis. Their results suggested that unlike BMP2 and BMP4, BMP7 induces the commitment of mesenchymal stem cells to the brown adipocyte lineage, thus leading to increased BAT mass [280]. Furthermore, a similar effect was observed in humans, although the experiments were performed only in isolated stem cells from human fat tissues [217]. BMP8B is another factor, which can promote brown adipocyte differentiation. Increased BMP8B expression is detected in BAT and the hypothalamus in response to cold exposure or high-fat feeding, which then activates BAT thermogenesis by activating the SMADs/p38 MAPK pathway in BATs and increasing the sympathetic tone to BAT [281].

#### 3.5.2. Peptidase M20 Domain Containing 1 (PM20D1)

The discovery of PM20D1 proposed a new concept of cold-induced thermogenesis. PM20D1 is preferentially expressed in UCP1+ adipocytes and can be secreted upon cold-stimulation. When PM20D1 is present in adipocytes, it generates a metabolite, N-acyl amino acid, which acts as an endogenous uncoupler in mitochondria, by condensation of fatty acids and amino acids, resulting in enhanced energy expenditure independently of UCP1 in adipocytes [282].

#### 3.5.3. Basic Fibroblast Growth Factor (bFGF/FGF2)

Cold acclimation in mice increased BAT mass with a remarkable increase in the expression of bFGF (or FGF2). Yamashita et al. suggested that increased bFGF levels expand the BAT mass by stimulating brown preadipocyte (adipogenesis) and vascular endothelial cell (vascularization) proliferation in an autocrine and paracrine manner, respectively [283].

#### 3.5.4. Wingless-Related MMTV Integration Site 10b (WNT10b)

WNT10b is a secretory protein and a member of the WNT family. Its significance was identified when induction of its activity in injured cardiac muscles showed a beneficial outcome by regulating coronary vessel formation in the heart. In the AT, WNT10b acts as key leverage in determining mesenchymal stem cell (MSC) fate between adipocyte and bone formation. Its expression inhibits adipocyte differentiation and is thus negatively associated with adipogenesis, but supports osteoblast differentiation in an autocrine or paracrine manner [284,285]. Furthermore, Rahman et al. identified that WNT10b is detected in beige WAT of bone marrow in response to energy state, and determines MSC fate [286].

#### 3.5.5. Insulin-Like Growth Factor-Binding Protein-2 (IGFBP2)

IGFBP2 is a member of the IGFBP family that regulates IGF-1 functions. This protein is involved in cellular processes, such as proliferation and migration, and links the communication between energy metabolism and bone formation. IGFBP2 was identified as a secretory protein by beige adipocytes [286]. However, its physiological significance is yet to be elucidated.

#### 3.5.6. Retinol-Binding Protein-4 (RBP4)

RBP4 is a transporter for vitamin A and its derivatives and is mainly synthesized in the liver [287]. RBP4 is also an adipokine, but its levels in the WAT appear to be negatively associated with insulin sensitivity [98]. In response to a β3-adrenergic cue or cold exposure, RBP4 is synthesized and released by the BAT. However, the amount of RBP4 released from the BAT contributes only to a negligible part in the total level of circulating RBP4. Further, the physiological significance of RBP4 from BAT remains unknown [288].

#### 3.5.7. Nerve Growth Factor (NGF)

A remarkable feature of BAT and beige AT development is the numerous sympathetic nerve innervations. It has been observed that the extent of nerve innervation is dynamically and flexibly altered in response to environmental cues, especially, cold temperature [289]. NGF is one of the factors being identified as a regulator for nerve innervation in BAT. Cold exposure in mice increases NGF synthesis in the BAT, which results in increased neurite outgrowth. In addition, treatment with anti-NGF serum blocks the NGF effect [290]. However, it still has to be elucidated whether NGF is effective enough in humans and in vivo.

## 4. Exosomal microRNAs as Novel Adipokines

### 4.1. Exosomes

Exosomes are extracellular nanovesicles that facilitate intercellular communication. They are actively released via the endosomal pathway and contain RNAs and proteins that can be transferred locally to adjacent cells or remotely to distant organs [291,292]. Proteins and RNAs delivered by exosomes can modulate the activities or properties of recipient cells, influencing diverse physiological and pathological functions such as proliferation and differentiation, immunomodulation, and tumorigenesis [293,294,295,296]. Recently, microRNAs (miRNA) were found in exosomes, and the physiological functions of the released miRNAs have started to gain attention [297,298,299].

### 4.2. MicroRNAs in the AT

MicroRNA is a small noncoding RNA of about 22nt, which binds to target mRNA and induces mRNA degradation and translational repression [300]. The role of miRNA in post-transcriptional control is critical in most cellular processes, and therefore, disorders in miRNA regulation are closely linked to human diseases [301,302,303]. The importance of miRNA in AT has been established through adipose-specific knockout of *Dicer* or *DGCR8*, the key components of miRNA biogenesis [304,305,306]. The loss of miRNA production results in defects of AT formation and metabolic dysregulation accompanied by insulin resistance and alterations of circulating lipids. Moreover, dozens of individual miRNAs have been reported to control fat metabolism, including the differentiation and function of white and brown adipocytes [307]. However, the function and abundance of miRNA have been analyzed mainly in terms of the autonomous role [308,309,310]. Exosomal miRNAs released from AT have newly emerged as novel players that regulate systemic metabolism by connecting different organs [311,312].

### 4.3. Adipose-Derived Exosomal microRNAs

#### 4.3.1. miR-99b

**Figure 5:**
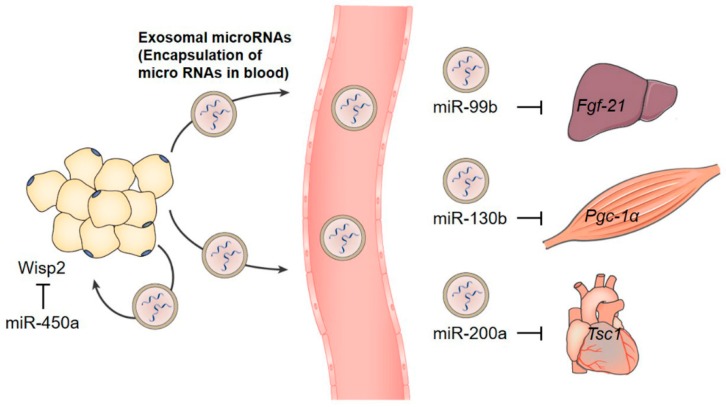
Adipose tissue-derived exosomal microRNAs. Recipient organs and target mRNAs of exosomal microRNAs. Brown fat-derived miR-99b suppresses *Fgf-21* expression in the liver, which regulates the systemic homeostasis. The circulating mIR-130b is correlated with BMI. In a mouse obesity model, adipose tissue-derived miR-130b downregulated *Pgc-1α* expression in the muscle cell. The expression and secretion of miR-200a are increased by rosiglitazone treatment. Exosomal miR-200a derived from adipocytes stimulates mTOR signaling by decreasing TSC1 expression, which leads to cardiomyocyte hypertrophy. Exosomal miR-450a functions in an autocrine manner to increase adipogenesis through the downregulation of WISP2. Abbreviations: FGF21, fibroblast growth factor 21; BMI, body mass index; PGC-1α, peroxisome proliferator–activated receptor gamma coactivator-1 alpha; mTOR, mammalian target of rapamycin; TSC1, tuberous sclerosis 1; WISP2, WNT1-inducible-signaling pathway protein 2.

Recently, Thomou et al. provided fascinating evidence suggesting that the miRNAs released by AT can regulate gene expression in distant metabolic organs [26] (Figure 5). They first found a strong reduction of exosomal miRNA in adipose-specific Dicer knockout (ADicerKO) mice, which blocks the production of miRNA in AT. The levels of 419 exosomal miRNAs were dramatically decreased in ADicerKO mice compared to wild-type mice. Transplanting the fat depot (brown fat, inguinal white fat, or epididymal white fat) from the wild type restored the miRNA level, suggesting that AT is a major source of circulating exosomal miRNAs. In humans, circulating miRNAs are also originated from the AT. A similar reduction was observed in congenital or HIV-associated lipodystrophy patients who suffer from a generalized loss of AT [313]; levels of 217 exosomal miRNAs were downregulated. Among 75 miRNAs decreased in the serum of both patient cohorts, 30 miRNAs were overlapped with the adipose-derived exosomal miRNAs downregulated in ADicerKO mice. Interestingly, Thomou et al. [26] also found a threefold higher level of circulating FGF21 in ADicerKO mice with a marked increase of *Fgf21* mRNA level in liver, muscle, pancreas, and fat. The transplantation of normal brown fat to ADicerKO mice decreased the level of *Fgf21* mRNA in liver, which comes with a reduction of circulating FGF21. Thomou et al. [26] identified miR-99b as a factor delivered from the AT to the liver for the regulation of FGF21 expression. MiR-99b is present in brown fat-derived exosomes and can bind the 3′UTR of *Fgf21* mRNA to inhibit the expression of FGF21. These results collectively suggest that miRNA secreted from the fat depots regulate gene expression in the liver, which in turn influence whole-body metabolism.

#### 4.3.2. miR-200a

The role of another exosomal miRNA released from AT has been reported by Fang et al. In the specific context of PPARγ activation, exosomal miR-200a was reported to mediate the crosstalk between adipocytes and cardiomyocytes in mice [314] (Figure 5). Treatment with rosiglitazone, a PPARγ agonist, activated the expression and secretion of miR-200a. Increased levels of exosomal miR-200a from adipocytes were delivered to cardiomyocytes to decrease TSC1 expression and subsequently stimulate mTOR signaling, leading to cardiomyocyte hypertrophy. Rosiglitazone, which is used to treat diabetes as an insulin sensitizer, has adverse cardiovascular effects [315]. The exosomal miR-200a-mediated inter-organ communication could be the molecular mechanism underlying the adverse effect of rosiglitazone.

#### 4.3.3. miR-450a

Exosomal miRNAs with autocrine functions have also been identified. Zhang et al. found an enriched population of 45 miRNAs in exosomes released from ATs (EXO-AT), compared to those released from adipose tissue-derived stem cells (EXO-ADSCs), in rats [316]. Among them, 14 miRNAs participated in the regulation of adipogenesis. In particular, miR-450a, one of the abundant miRNAs, 7-fold enriched in EXO-AT compared to EXO-ADSC, increased adipogenesis by inhibiting WISP2 (Figure 5).

#### 4.3.4. miR-155

Exosomal microRNA originating from macrophages in AT has been proven to modulate systemic metabolism [317]. Ying et al. [317] reported that AT macrophages (ATMs) secrete miRNA-containing exosomes. They also showed that ATM-derived exosomes in obese mice induce glucose intolerance and insulin resistance when injected into lean mice, and conversely ATM-derived exosomes in lean mice improve insulin sensitivity when injected into obese mice [317]. This finding suggests that ATM-derived exosomes serve to transfer the critical regulator of glucose homeostasis and insulin sensitivity to metabolic target cells of liver and muscle. There were 20 miRNAs with significant differences in abundance between lean and obese ATM-derived exosomes, among which miR-155 overexpressed in obese ATM exosomes was found to suppress insulin action on glucose production through the downregulation of its target, PPARγ mRNA.

### 4.4. Clinical Research on Exosomal microRNAs in Metabolic Disorders

Clinical studies have started to explore exosomal microRNAs derived from AT, especially in relation to obesity and associated metabolic disorders [25,318,319]. The abundance of several adipocyte-derived exosomal miRNAs was altered in obese patients, and most of them were specific to the VAT or sWAT. Exosomal miRNAs derived from VAT were predicted to target mRNAs mainly involved in TGF-β and Wnt/β-catenin signaling [25,320]. Another study on circulating miRNAs in obese patients showed that miR-130b in serum was positively correlated with BMI (Figure 5). The role of exosomal miR-130b was further defined in a mouse obesity model where the secretion of miR-130b, stimulated by TGF-β in adipocytes, targeted PGC-1α mRNA in muscle cells [318]. In a study with obese patients who underwent gastric bypass bariatric surgery, the relationship between adipocyte-derived exosomal miRNAs and insulin resistance was verified [319]. Bariatric surgery that leads to weight loss and improved glucose regulation also modified adipocyte-derived exosomal miRNAs. Fifty-six miRNAs were differentially expressed one year after surgery, and putative targets of these miRNAs were enriched in insulin receptor signaling. The change in abundance of 46 miRNAs was significantly correlated to the change in insulin resistance. In addition to the adipose-derived exosomal miRNAs, circulating miRNAs whose origins were not defined, have been increasingly reported in numerous clinical research related to metabolic disorders [321,322,323,324,325,326]. Further characterization of circulating miRNAs may further expand our knowledge of the specific relationship between adipose-derived miRNAs and metabolic diseases.

### 4.5. Challenges and Perspectives as Diagnostic and Therapeutic Tools

As reviewed herein, accumulated studies on miRNAs secreted from the AT have identified exosomal miRNAs as a genetic regulator form of adipokines. Exosomal microRNAs reflect the physiological property of the origin and alter the target cell by regulating the expression of the target gene. Furthermore, inter-organ communication by adipose tissue-derived exosomal miRNAs alters the systemic metabolic profile. These miRNAs could be developed as diagnostic markers of metabolic diseases, and may be used to develop novel therapeutic strategies [327]. However, detailed mechanisms on the transfer of miRNAs via exosomes remain to be elucidated, including detailed mechanisms of how miRNAs are selectively loaded into exosomes and how exosomes target specific organs or cell types. An improved understanding of the specificity of exosomal miRNA in both production and action would unveil another layer of regulation on systemic metabolism.

## 5. Discussion

ATs, as endocrine organs, secrete peptides or proteins called “adipokines” for the maintenance of energy homeostasis. Recently, it was reported that most physiologically active substances produced by ATs can act as adipokines. Adipokines are not limited to the secretome derived from WAT. Batokines and exosomal microRNAs also belong to the broader family of adipokines. Batokines and exosomal microRNAs derived from ATs act like hormones and affect other metabolic organs in an endocrine fashion. In particular, exosomal miRNA has been in the spotlight, as a new target for a therapeutic and diagnostic biomarker for obesity-related diseases.

Changes in the expression pattern of these adipokines mirror the AT status, and an altered secretion pattern of adipokines is frequently observed in metabolic diseases such as obesity and T2DM. Adipose-derived factors are closely related to insulin resistance, adiposity, inflammation, and other pathogenic conditions. Therefore, observing changes in adipose-derived factors is very important for a better understanding of the underlying pathogenesis and pathophysiology of metabolic diseases. It will enable us to diagnose and predict obesity and T2DM. Further, it will support the development of adipose-derived factors as biomarkers and targets for therapeutic intervention and pharmacotherapeutic management of obesity and T2DM.

In contrast, further studies on the metabolic effects of administration of adipokines as a therapeutic drug are necessary. Administration of adipokines could trigger unexpected effects, owing to the different physiological and pathological conditions in humans. Additionally, the effect could be different in rodents and humans.
